# A Large Grade 5 Mobile Aortic Arch Atheromatous Plaque: Cause of Cerebrovascular Accident

**DOI:** 10.1155/2018/5134309

**Published:** 2018-04-01

**Authors:** Chikezie Alvarez, Hafiz Muhammad Aslam, Sara Wallach, Muhammad U. Mustafa

**Affiliations:** ^1^Seton Hall University, St. Francis Medical Center, Trenton, NJ, USA; ^2^Capital Health Center, Hamilton, NJ, USA

## Abstract

Aortic atheromas (aortic atheromatous plaques) are defined by an irregular thickening of the intima ≥2 mm, and a complex plaque is defined as a protruding atheroma ≥4 mm with or without an attached mobile component. Stroke incidence is approximately 25% in patients with mobile plaques of the aortic arch and 2% in patients with quiescent nonmobile plaques. Antiplatelet agents, oral anticoagulants, and statins have been suggested in the management of atheromas. We present an 80-year-old male, with non-ST-segment elevation myocardial infarction (NSTEMI) and chronic dysarthria, found to have an acute cerebrovascular accident (CVA) secondary to embolism from a large 12 mm aortic arch plaque, treated medically with oral antiplatelet therapy, anticoagulation, and statin therapy.

## 1. Introduction

Aortic atheromas pose a significant risk for CVA due to microembolization. The presence or absence of calcification has a greater impact on embolization risk because the lack of calcification makes atheroma more lipid-laden and thus more prone to ulceration, thrombosis, and rupture [1]. Aortic arch atheroma and plaque complexity are important risk factors for unexplained arterial embolic events such as stroke, transient ischemic attack (TIA), and peripheral emboli [2]. Stroke incidence is around 25% in patients with mobile plaques of the aortic arch, while it is only 2% in patients with quiescent nonmobile plaques [3]. Risk factors independently associated with complex plaque formation are advanced age, history of HTN, hypercholesterolemia, increased BMI, diabetes, and tobacco use [4]. Transesophageal echocardiogram (TEE) is a safe and relatively less invasive procedure and is the modality of choice for diagnosing an aortic atheroma. This enables a detailed view of the aorta and quantification of atheromatous plaque according to their thickness and presence of mobile components [5–7].

## 2. Case Presentation

An 80-year-old male with a past medical history of HTN, DM-II, mild vascular dementia, bilateral cerebrovascular accident with no residual weakness, gout, and osteoarthritis presented to the hospital with chest pain and was found to have a non-ST elevation myocardial infarction (NSTEMI) with a peak troponin of 5.1. The patient also had an episode of slurred speech in the ER for which Neurology was consulted. Upon further investigation, his slurred speech was chronic in nature. Home medications included aspirin 81 mg, allopurinol, amlodipine, pantoprazole, and glipizide. Vital signs on admission revealed a blood pressure of 163/98 mmHg without orthostasis, respiration rate of 19, heart rate of 87 beats per minute, and a temperature of 98.2° F. Physical examination revealed no carotid bruits, lungs were clear, no JVD, and cardiovascular examination was unremarkable: normal S1/S2, no murmurs, rubs, or gallops. On neurological examination, the patient was alert and oriented to person, place, and time with mild dysarthria. The National Institutes of Health (NIH) stroke scale score was 1. Cranial nerves II–XII were grossly intact, and stretch reflexes were 2+ throughout. Tactile sensation was intact, and motor function was 5/5 throughout with no cerebral ataxia present. At the time of admission, his electrolytes were within normal limits; however, BUN and Cr were 1.76 and 27 and iron deficiency anemia was present (HB: 11.8, HCT: 37.3, MCV: 88.5, RDW: 17.6, ferritin: 200, iron: 22, TIBC: 220, and transferrin: 176). EKG showed normal sinus rhythm with nonspecific T wave abnormalities. Serum lipids and uric acid were normal in range. CXR was negative for effusion, consolidation, and pneumothorax. The patient was started on heparin drip and was transferred to another facility for cardiac catheterization, which revealed a mid-LAD obstructive lesion, which was treated with a drug-eluting stent. The patient was started on dual antiplatelet therapy (DAPT) with aspirin 81 mg and Plavix 75 mg.

CT head without contrast revealed no acute intracranial hemorrhage with encephalomalacia and gliosis in the right frontal and occipital lobes, in keeping with prior infarctions.

**Figure 1:**
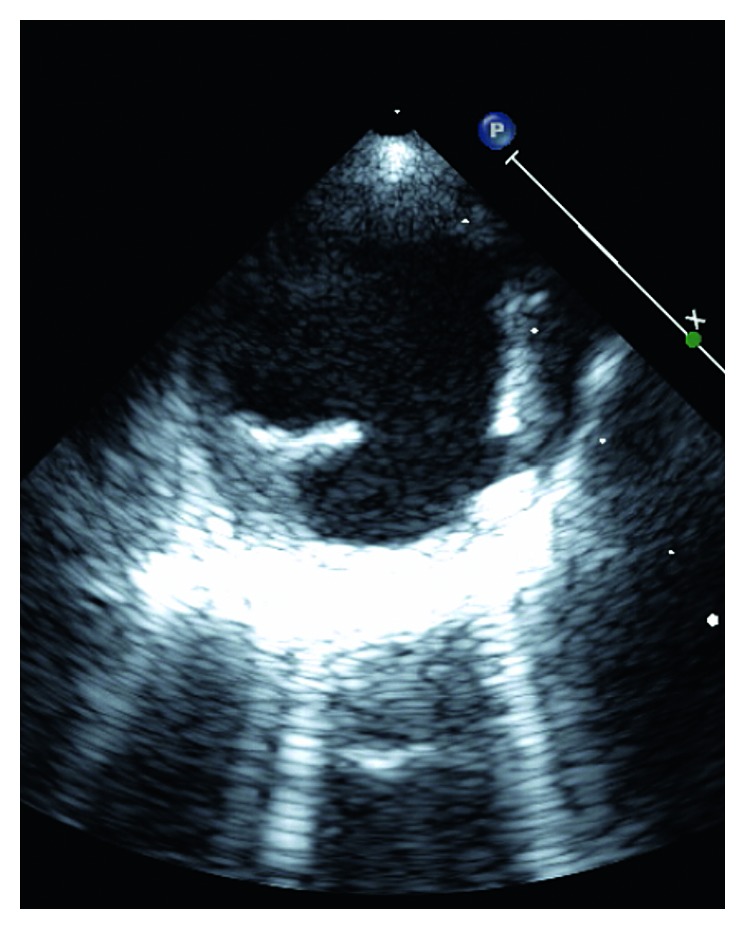
Short-axis TEE view of atheromatous aortic plaque.

**Figure 2:**
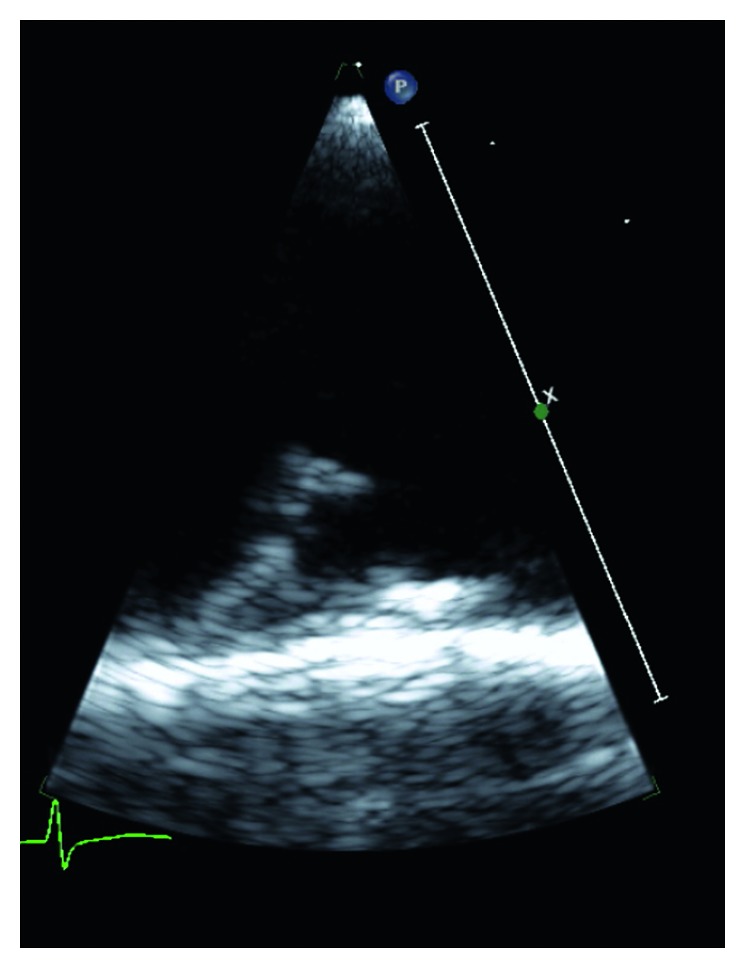
Long-axis TEE view revealing mobile atheromatous plaque.

**Figure 3:**
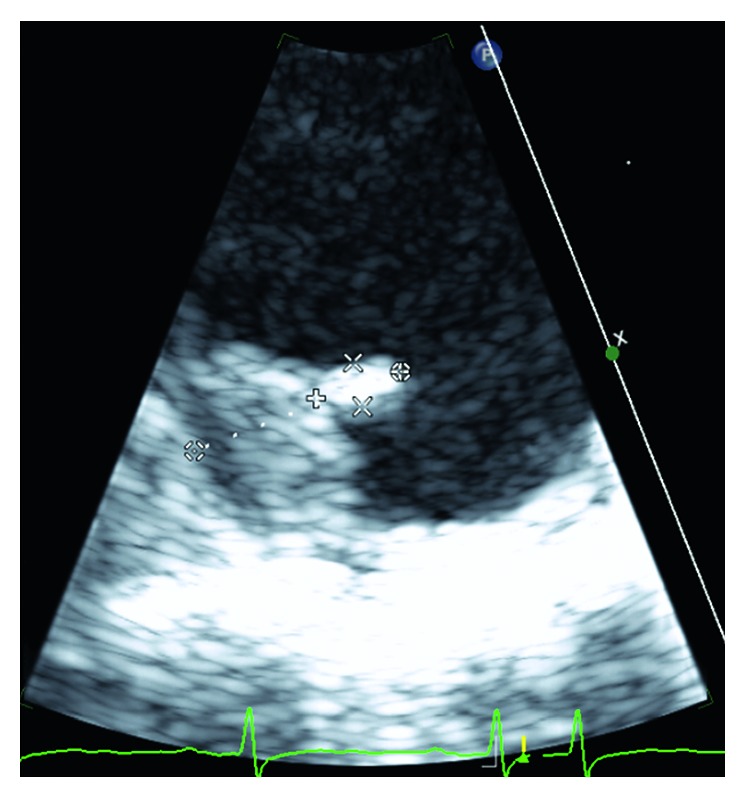
Short-axis TEE view with 12 mm mobile aortic arch plaque.

**Figure 4:**
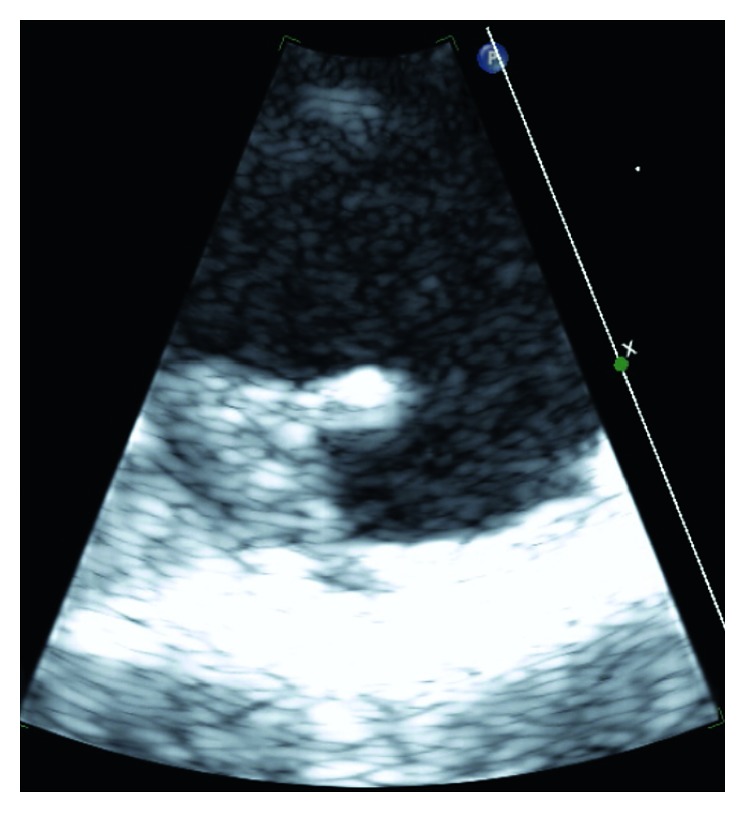
Magnified short-axis aortic view of 12 mm aortic arch plaque.

MRI brain revealed a small foci of restricted diffusion in the left cerebellum, right periventricular, and left corona radiata consistent with small acute infarcts, likely embolic in origin. There were old cortical infarcts in the right frontal and occipital lobes, with remote lacunar infarcts in the bilateral basal ganglia, thalami, and bilateral cerebellar hemispheres. Transesophageal echocardiogram (TEE) revealed an ejection fraction (EF) of 55–60% with aortic root and vascular sclerosis. It also revealed mild dilation of the aortic root with a visualized plaque in the transverse aorta. There was evidence of a Grade 5 (mobile or ulcerating) atheroma in the transverse aorta, with a visualized plaque in the descending aorta. There was also evidence of a Grade 4 (>5 mm) atheroma in the descending aorta. A severe mobile atherosclerotic plaque was noted in the aortic arch, which was approximately 12 mm long (Figures 1–4). There was no patent foramen ovale.

The patients' family decided against surgical management, and he was eventually discharged on conservative management with aspirin 81 mg, Plavix 75 mg, Lipitor 80 mg, Lopressor, and apixaban 5 mg BID for anticoagulation.

## 3. Discussion

Aortic atheromas can be classified as stable and unstable with unstable being mobile, nonhomogeneous, ulcerated, or spongiform, while stable plaques are calcified, immobile, echo dense homogeneous, and without signs of ulceration. The incidence of an aortic plaque is approximately 45% and can result in an embolism [1]. Aortic atheromas can occur in various regions of the aorta, while in ascending aorta and arch, it is extremely rare. The natural history of aortic atheromas is still unknown and diagnosis is often made only after a thromboembolic event, which can be life threatening [8]. The presence of large plaques in the proximal segment of aorta is a main risk factor of stroke, and studies have shown that it can result in a 2.5- to 4-fold increased risk of stroke [9, 10].

Although many studies have investigated aortic atheromas, the mechanism of thrombus formation remains unclear. Pagni et al. and Lohrmann and Peters investigated the causes of aortic atheromas, and their studies found that the cause may be a hypercoagulable state, deficiency in one or more components of coagulation-anticoagulation system, secondary to underlying lupus, estrogen therapy, heparin immune reaction, or underlying aortic disease [11]. Some authors, however, have reported aortic atheromas without evidence of any of the aforementioned conditions [11, 12]. In our patient, there was no evidence of a hypercoagulable or systemic disorder that would cause unusual thrombosis, but there was an atherosclerotic plaque, which could result in endothelial injury and resulting thrombus formation.

TEE is a safe, noninvasive, and modality of choice for diagnosis, although CT, MRI, and intraoperative epiaortic ultrasonography are known to be complementary examination techniques. TEE was able to find aortic arch atheromatous disease in 55% of patients with a normal chest X-ray, and 91% of those who had heavily calcified aortic knobs [13]. TEE has a sensitivity of 91%, specificity of 82%, and positive and negative predictive values of 72% and 95%, respectively [13, 14]. The Katz classification for grading aortic atheromas is as follows: Grade 1, normal-appearing intima of the aorta; Grade 2, extensive intimal thickening; Grade 3, sessile atheroma protruding <5  mm into the aorta; Grade 4, atheroma protruding >5  mm; and Grade 5, mobile atheroma [15].

In our patient, TEE revealed the presence of atherosclerotic plaques with intimal disruption and mobile projections in the aortic arch, while no potential embolic sources were detected in the extracranial carotid or vertebral arteries. We can conclude that the ulcerated plaque with mobile projections were likely responsible for the cerebral emboli found in our patient. In previous studies, it was reported that when atherosclerotic plaques become pedunculated and highly mobile, the incidence of an embolic event is high [16]. In this present case, the atherosclerotic plaques were pedunculated and highly mobile, and we could not find any other cause of cerebral embolism.

Studies have shown that among patients with ischemic stroke who are treated with warfarin or aspirin, large aortic plaques and especially those with complex morphology remained associated with an increased risk of recurrent stroke and death [17]. Regarding antithrombotic therapy, there have been a limited number of randomized control trials which have compared antiplatelet therapy to anticoagulant therapy with mixed results. The Aortic Arch Related Cerebral Hazard (ARCH) trial which randomized patients to aspirin plus clopidogrel or warfarin in patients with an aortic arch plaque > 4 mm found that ischemic stroke occurred less frequently in the aspirin plus clopidogrel group; however, these results were statistically insignificant [18]. Other studies have found no significant benefit to single antiplatelet therapy over dual antiplatelet therapy with aspirin plus clopidogrel [19–21]. The 2014 American Heart Association/American Stroke Association (AHA/ASA) guidelines recommend antiplatelet therapy and statin treatment for patients with an ischemic stroke and evidence for an aortic arch atheroma [22]. Currently, the role of surgical intervention to treat patients with aortic plaques is not clearly defined. Although studies with endovascular treatment with angioplasty or stenting have been reported, concerns about destabilizing atheromatous material with catheter manipulation are well founded [23]. Aortic arch atherectomy may be limited to patients who are also undergoing cardiac surgery; however, this is not without an intraoperative risk of stroke [23]. The AHA/ASA recommends against routine surgical endarterectomy of aortic arch plaque for the purposes of secondary stroke prevention [22].

Our patient was discharged on dual antiplatelet therapy with aspirin plus clopidogrel and apixaban for anticoagulation. Although non-vitamin-K oral anticoagulants have not been extensively studied for secondary ischemic stroke prevention in patients with an aortic atheroma >4 mm; our patient on follow-up did not have any subsequent cerebrovascular events or any episodes of major bleeding.

## 4. Conclusion

Atheromas with >4 mm thickness or with plaque rupture and mobile fragments are more likely to be associated with peripheral embolic events. Antithrombotic agents and statins remain the mainstay of treatment, while the role and benefit of surgical intervention are not well established and therefore currently not recommended. The use of non-vitamin-K oral anticoagulants is another avenue for future investigation.
